# Cadet De Gassicourt

**Published:** 1822-02

**Authors:** 


### CADET DE GASSICOURT.

This distinguished literary character and *pharmacien*, whose practical knowledge of the natural sciences, medicine, and chemistry, was only equalled by the variety and utility of his other numerous talents, —whose works have been often translated, quoted, and commented upon, by the scientific men of every nation in Europe,—died on the 21st of November, 1820, aged 56 years, after a very severe illness, which he bore with exemplary fortitude and resignation.

